# Evolutionary and functional dynamics of a leishmanolysin-like immune multigene family during early infection in *Philasterides dicentrarchi*

**DOI:** 10.3389/fimmu.2026.1864431

**Published:** 2026-07-07

**Authors:** Paola Gulias, Jesús Lamas, Rosa Ana Sueiro, Verónica Blanco-Abad, Alejandro Cés, José Manuel Leiro Vidal

**Affiliations:** Laboratory of Parasitology and Immunobiology, Institute for Research on the Aquatic Environment for Global Health (iARCUS) and Health Research Institute of Santiago de Compostela (IDIS), Universidad de Santiago de Compostela (USC), Santiago de Compostela, Spain

**Keywords:** host–parasite interaction, immune evasion, leishmanolysin, metalloproteases, multigene family, *Philasterides dicentrarchi*, proteomics, RNA-Seq

## Abstract

**Introduction:**

The evolutionary dynamics of multigene families are central to shaping host–parasite interactions and immune outcomes. In parasitic protists, expansion and diversification of surface-associated gene families enable immune evasion and host modulation, yet these mechanisms remain poorly understood in marine ciliates. *Philasterides dicentrarchi*, the causative agent of scuticociliatosis, provides a relevant model to investigate how gene family evolution contributes to parasite virulence and immune interaction.

**Methods:**

We performed an integrated multi-omics analysis combining genome-wide screening using Hidden Markov Models, repeat annotation, phylogenetic inference, RNA-seq-based transcriptomic profiling, and LC–MS/MS analysis of the surface-associated fraction. Structural features of leishmanolysin-like (LSF) proteins were predicted using domain and membrane-targeting analyses, and expression dynamics were evaluated across early infection time points.

**Results:**

Genome-wide analysis identified 16 annotated LSF genes and a broader repertoire of 73 LSF-related sequences, consistent with a dynamic birth–death model of multigene family evolution. This expansion was associated with a repeat-rich genomic landscape dominated by simple and low-complexity elements, suggesting that genome plasticity contributes to diversification. LSF proteins retained conserved M8 metalloprotease domains and membrane-targeting features but displayed substantial sequence and regulatory divergence. Transcriptomic data revealed rapid and coordinated upregulation at 1 h post-infection, followed by a decline at later time points. Proteomic analysis confirmed the presence of most LSF proteins at the parasite surface and demonstrated a positive correlation between transcript abundance and protein detection.

**Conclusion:**

Our integrative multi-omics analysis reveals that the leishmanolysin-like family (LSF) of *Philasterides dicentrarchi* is a highly expanded and diversified multigene repertoire. The rapid induction of LSF transcripts during early infection and the detection of most family members in the surface-associated proteome indicate their active deployment at the host–parasite interface. These findings provide new insights into the evolutionary and functional dynamics of immune-interacting multigene families in marine parasitic ciliates.

## 1 Introduction

Host–parasite interactions are shaped by a dynamic evolutionary arms race in which parasites continuously adapt to host immune defences, while hosts evolve mechanisms to detect and eliminate invading pathogens (1). A central feature of this co-evolutionary process is the expansion and diversification of multigene families encoding immune-interacting molecules, which provide functional redundancy and enable rapid adaptation to fluctuating immune environments. Such families are particularly relevant in protozoan parasites, where they often encode surface-exposed proteins involved in host recognition, immune evasion, and intracellular survival (2, 3). Among these, leishmanolysin-like metalloproteases (GP63 family) represent one of the best-characterized virulence systems in protozoa (4, 5). These zinc-dependent proteases are typically anchored to the parasite surface and have been shown to play critical roles in host–parasite interactions (6). In *Leishmania* spp., GP63 contributes to immune evasion by cleaving complement components such as C3b, thereby preventing complement-mediated lysis and facilitating parasite survival. In addition, GP63 promotes parasite internalization into macrophages through opsonin-mediated pathways and modulates host signaling by targeting key immune molecules, including regulators of oxidative burst, antigen presentation, and intracellular trafficking (7–9). Beyond complement evasion, GP63 has also been implicated in the manipulation of host cell signaling and intracellular trafficking, further supporting its role as a multifunctional immune-modulatory factor (9). More broadly, surface molecules of protozoan parasites constitute a highly specialized interface that mediates adhesion, immune recognition, and immune evasion (10). These molecules, including metalloproteases, glycoconjugates, and membrane-associated proteins, interact with host receptors such as complement receptors, Toll-like receptors, and Fc receptors, thereby actively shaping immune recognition, activation, and evasion processes (11). Importantly, many of these molecules are encoded by expanded multigene families, suggesting that gene duplication and diversification are key evolutionary strategies to modulate host immune responses.

Despite extensive knowledge in kinetoplastids, the role of leishmanolysin-like proteases in ciliates remains poorly understood (12). The scuticociliate *Philasterides dicentrarchi* is an emerging pathogen in marine aquaculture, responsible for severe systemic infections in fish (13–16). Previous studies have highlighted the importance of surface-associated antigens and proteases in parasite virulence and host immune recognition (17–21; Gulias et al., 2026). In particular, the identification of surface antigen fractions (SAF) enriched in immunoreactive proteins suggests that surface-exposed molecules play a central role in mediating host–parasite interactions and may represent key targets of the host humoral response, including complement-dependent cytotoxicity and antibody-mediated recognition (17–21). In this context, immune multigene families represent a fundamental mechanism shaping host–parasite interactions and evolutionary adaptation.

Recent advances in high-throughput sequencing and proteomics have enabled the simultaneous characterization of gene expression and protein deployment, providing new opportunities to investigate the functional dynamics of multigene families. In this context, multi-omics approaches combining transcriptomics and proteomics are particularly powerful to link gene expression with protein-level validation, thereby overcoming limitations associated with transcript-only analyses (22). Such integrative strategies are essential to identify which members of a multigene family are not only transcriptionally regulated but also functionally deployed at the parasite surface during infection.

In the present study, we investigate the evolutionary and immunological dynamics of a leishmanolysin-like multigene family (LSF1–LSF16) in *Philasterides dicentrarchi*. By integrating phylogenetic, transcriptomic, and surface proteomic data, we characterize the temporal regulation of these proteases during early infection and assess their deployment at the host–parasite interface. Our results reveal a coordinated transcriptional program coupled with proteomic evidence of surface localization, supporting a model in which this multigene family contributes to immune evasion and modulation of host immune responses during the initial stages of infection.

## 2 Materials and methods

### 2.1 Fish maintenance and ethical statement

Thirty juvenile turbot (*Scophthalmus maximus* L.), with an average body weight of approximately 20 g, were obtained from local commercial fish farms and were maintained in three 250-L tanks (10 fish per tank) supplied with aerated recirculating seawater at 16 °C, and were fed daily with commercial pellets (Skretting, Burgos, Spain). Fish were randomly distributed among tanks and maintained under identical husbandry conditions throughout the acclimation period. Animals were acclimated to laboratory conditions for two weeks prior to the start of the experimental procedures. Before experimental manipulation, fish were anesthetized by immersion in tricaine methane sulfonate (MS-222; Merck, Spain) dissolved in seawater before vaccination (60 mg L^-^¹) and prior to euthanasia (100 mg L^-^¹), which was performed by pithing.

All experimental procedures carried out in this study complied with European legislation (Directive 2010/63/EU) and Spanish regulations concerning the use of animals for scientific purposes (Royal Decree 53/2013). The experimental protocols were approved by the Institutional Animal Care and Use Committee of the University of Santiago de Compostela (Spain) (authorization number: 15012/2022/008).

### 2.2 Parasites and experimental infections

Trophonts of *Philasterides dicentrarchi* strain I1 were maintained under axenic laboratory conditions in sterile Leibovitz L-15 medium supplemented with 10% heat-inactivated fetal calf serum (FCS). Cultures were incubated at 18 °C and maintained in exponential growth phase by periodic subculturing.

For all experiments, trophonts were harvested during the logarithmic growth phase, collected by low-speed centrifugation (800 × g, 10 min, 4 °C), and washed three times in sterile phosphate-buffered saline (PBS, pH 7.4) to remove residual medium and debris. Unless otherwise indicated, all experiments were performed using independent biological replicates.

For transcriptomic analyses, fish were intraperitoneally injected with 100 µL of a suspension containing 1 × 10^7^ trophonts mL^-^¹. Trophonts maintained under axenic culture conditions were used as the 0 h control. Independent groups of three fish were sampled at 1, 2, and 4 h post-infection, generating three biological replicates per condition. Ciliates were recovered from the peritoneal cavity using a syringe fitted with an 18G hypodermic needle, washed twice in sterile Leibovitz L-15 medium at 4 °C, and processed for genomic DNA and total RNA extraction.

Trophonts recovered from each fish were processed independently for RNA extraction and subsequent transcriptomic analyses. Therefore, each RNA-seq library represented a biological replicate derived from a single fish. Fish were anesthetized prior to sampling, and all procedures were performed under aseptic conditions.

Experimental infection was performed to expose trophonts to host-derived immune factors and physiological conditions that cannot be reproduced under axenic culture conditions, thereby allowing the characterization of LSF expression during the earliest stages of host–parasite interaction.

### 2.3 Isolation and biotinylation of surface-associated proteins and LC–MS/MS analysis

Enrichment of surface-associated proteins was assessed by comparative LC-MS/MS analysis of biotinylated and non-biotinylated fractions. The relative depletion of abundant intracellular proteins, including actin-like and metabolic proteins, was used as an indicator of successful enrichment. Due to the limited amount of purified material obtained from ciliates, additional Western blot validation was not performed.

Surface-associated proteins were isolated using the Pierce™ Cell Surface Protein Isolation Kit (Thermo Fisher Scientific, Waltham, MA, USA; Supplier No. A44390), following the manufacturer’s instructions with minor modifications adapted for ciliates. This method is based on the selective labeling of extracellular proteins with the membrane-impermeable reagent Sulfo-NHS-SS-Biotin, followed by affinity purification of biotinylated proteins using immobilized NeutrAvidin resin. The procedure enables enrichment of proteins exposed at the cell surface while minimizing contamination from intracellular components. Briefly, 1 x 10^6^ freshly harvested trophonts were washed three times in ice-cold PBS and incubated with Sulfo-NHS-SS-Biotin (membrane-impermeable) for 30 min at 4 °C under gentle agitation to label extracellular lysine residues. Excess reagent was quenched according to the manufacturer’s protocol. Parallel non-biotinylated control samples were processed in parallel to evaluate non-specific binding during affinity purification. Enrichment of surface-associated proteins was further assessed by comparing the protein composition of biotinylated and non-biotinylated fractions and by monitoring the relative depletion of abundant cytosolic proteins. Cells were lysed using the provided lysis buffer supplemented with protease inhibitors, and lysates were clarified by centrifugation (10,000 × g, 10 min, 4 °C). Biotinylated proteins were affinity-purified using NeutrAvidin agarose resin, extensively washed, and eluted under reducing conditions using SDS sample buffer containing dithiothreitol (DTT).

Peptides were analyzed by nanoLC–MS/MS using a timsTOF Pro 2 mass spectrometer (Bruker Daltonics, Bremen, Germany) equipped with a CaptiveSpray nano-electrospray ionization (nanoESI) source and coupled to a nanoElute 2 nanoUHPLC system (Bruker Daltonics). Raw MS/MS data were processed using PEAKS Studio version 12.0 (Bioinformatics Solutions Inc., Waterloo, Canada) and searched against a custom *Philasterides dicentrarchi* protein database (*Philasterides*_NCBI_2026_05_04).

Database searches were performed using trypsin as the proteolytic enzyme with a maximum of two missed cleavages and semi-specific digestion mode. Parent mass tolerance was set to 15 ppm and fragment mass tolerance to 0.02 Da. Carbamidomethylation of cysteine residues was specified as a fixed modification, whereas protein N-terminal acetylation, deamidation (N/Q), dehydration, and methionine oxidation were considered variable modifications. A maximum of three variable post-translational modifications per peptide was allowed.

Peptide identifications were filtered using a false discovery rate (FDR) of 1%, and proteins were accepted when achieving a minimum protein score of −10LgP ≥ 20. Surface enrichment was evaluated by comparing biotinylated and non-biotinylated fractions and by monitoring the relative abundance of representative intracellular proteins in LC–MS/MS datasets. Identified peptides were mapped against a curated dataset of leishmanolysin-like proteins (LSF1–LSF16) to assess their representation within the surface proteome.

### 2.4 DNA and RNA extraction, sequencing, and *de novo* assembly

Trophonts were collected during exponential growth, washed extensively, and processed immediately to minimize degradation and contamination.

Genomic DNA was extracted from *Philasterides dicentrarchi* trophonts using the PureLink™ Genomic DNA Mini Kit (Invitrogen™, Thermo Fisher Scientific, USA; Cat. No. K182001) according to the manufacturer’s instructions. Briefly, trophont pellets were lysed using the kit lysis buffer and Proteinase K, followed by purification through silica-based spin columns. After successive washing steps to remove proteins, salts, and other contaminants, genomic DNA was eluted in nuclease-free water and stored at −20 °C until further use.

DNA concentration and purity were determined spectrophotometrically using a NanoDrop™ instrument (Thermo Fisher Scientific, USA). DNA integrity was assessed by electrophoresis in 1% agarose gels stained with an appropriate nucleic acid dye. Only high-quality DNA samples exhibiting A260/A280 ratios between 1.8 and 2.0 and showing no evidence of degradation were used for downstream molecular analyses. Purified genomic DNA was subsequently used for whole-genome sequencing and genome assembly analyses.

Total RNA was extracted from *P. dicentrarchi* trophonts previously preserved in TRIzol™ Reagent (Invitrogen, Thermo Fisher Scientific, USA) and stored at −80 °C until processing. Approximately 10^6^ ciliates were suspended in 1 mL of TRIzol™ Reagent and thawed on ice. Following complete homogenization, 200 μL of chloroform were added per milliliter of TRIzol™, and samples were vigorously shaken for 15 s, incubated for 2–3 min at room temperature, and centrifuged at 12,000 × g for 15 min at 4 °C. The aqueous phase was carefully recovered and mixed with an equal volume of 70% ethanol.

RNA purification was performed using the PureLink™ RNA Mini Kit (Invitrogen, Thermo Fisher Scientific, USA) according to the manufacturer’s instructions for TRIzol-derived samples. Residual genomic DNA was removed by on-column digestion using the PureLink™ DNase Set (Invitrogen, Thermo Fisher Scientific, USA). Briefly, 10 μL of reconstituted DNase I were mixed with 70 μL of DNase I Reaction Buffer and applied directly to the membrane. After incubation for 15 min at room temperature, the columns were washed according to the manufacturer’s instructions. Purified RNA was eluted in RNase-free water and stored at −80 °C until further analysis.

RNA concentration and purity were determined using a NanoDrop™ spectrophotometer (Thermo Fisher Scientific, USA) and a Qubit™ RNA High Sensitivity Assay Kit (Thermo Fisher Scientific, USA). RNA integrity was assessed using an Agilent 2100 Bioanalyzer (Agilent Technologies, USA), and only high-quality RNA samples were used for library preparation. Sequencing libraries were prepared using Illumina-compatible protocols and sequenced in paired-end mode. Raw reads were quality-checked using FastQC and trimmed using fastp to remove low-quality bases and adapter sequences.

To minimize host contamination, filtered reads were aligned against the *Scophthalmus maximus* reference genome using Bowtie2 (v2.5.4) (23), and only unmapped reads were retained.

*De novo* transcriptome assembly was performed using Trinity (24), and genome assembly was carried out using SPAdes (k-mer sizes: 21, 33, 55, 77) (25). Assembly quality was assessed using QUAST. Assembly statistics, including transcript number, transcript length distribution, and N50 values, were calculated from the final Trinity assembly and used to evaluate transcriptome completeness and contiguity.

Transcript abundance was quantified using Salmon (26), and expression values were reported as transcripts per million (TPM). Open reading frames were predicted using TransDecoder with the ciliate genetic code.Transcriptomic analyses were performed using biological triplicates for each infection time point. Trophonts of *Philasterides dicentrarchi* were recovered at 0 h (control), 1 h, 2 h, and 4 h post-infection, generating a total of 12 biological samples. Total RNA extracted from each sample was used for the preparation of Illumina RNA-seq libraries. Libraries were sequenced by ZF-Genomics (Leiden, The Netherlands) using an Illumina HiSeq2500 platform. During library quality control, two libraries corresponding to the 4 h post-infection condition failed to generate sufficient sequencing material and were excluded from downstream analyses. Consequently, transcriptomic analyses were performed using ten libraries representing the four infection stages.

### 2.5 Functional annotation and multi-omics integration

Predicted protein sequences were annotated using BLASTp searches against the NCBI non-redundant (nr) database (E-value < 1e^-5^), and domain architecture was characterized using InterProScan (27). Redundant sequences were collapsed, and manual curation was performed for protein families of interest. Integration of genomic, transcriptomic, and proteomic datasets provided complementary evidence supporting candidate protein annotation and identification across multiple molecular levels, ensuring robust annotation of surface-associated proteins and multigene families. Because the objective of this study was focused on the evolutionary diversification, structural characterization, and temporal deployment of a predefined leishmanolysin-like gene family rather than on genome-wide differential expression analyses, Gene Ontology (GO) and KEGG enrichment analyses were not performed.

### 2.6 Identification of leishmanolysin-like proteins (LSF)

LSF candidates were identified using a combination of BLAST-based homology searches and Hidden Markov Model (HMM) profiling against metalloprotease databases. Sequences were retained only if they contained conserved features of the M8 metalloprotease family, including the HEXXH catalytic motif. Structural characteristics such as cysteine-rich regions, domain organization, and sequence length were also considered during candidate selection. Sequence redundancy was reduced using clustering approaches, and candidate LSF sequences were further supported by transcriptomic and genomic evidence, while proteomic data were used to assess protein-level expression. No minimum sequence-length threshold was imposed; candidates were retained based on the presence of conserved M8 metalloprotease signatures together with supporting transcriptomic, genomic, and proteomic evidence.

### 2.7 Prediction of signal peptides, GPI anchors, and structural features

Signal peptides were predicted using SignalP (28), whereas GPI-anchor signals were identified using PredGPI and big-PI Predictor. Transmembrane domains were predicted using TMHMM, and proteins containing both signal peptides and GPI-anchor signals were classified as putative surface-associated proteins. Additional structural features, including cysteine-rich regions, conserved domains, and overall protein architecture, were evaluated using InterProScan and manual inspection to further support functional annotation and subcellular localization predictions.

### 2.8 Phylogenetic analysis

Protein sequences were aligned using MAFFT and refined using TrimAl to remove poorly aligned regions. Phylogenetic trees were reconstructed using IQ-TREE (29) with ModelFinder (30) for automatic model selection. Branch support was assessed using ultrafast bootstrap (1,000 replicates) and SH-aLRT tests. Trees were visualized and edited using FigTree. Due to the limited amount of purified material and the absence of specific antibodies against individual LSF proteins, additional immunoblot validation was not performed.

### 2.9 Statistical analysis

RNA-seq data were analyzed using TPM values for descriptive and comparative purposes. Correlation between transcript abundance and proteomic detection was assessed using Spearman’s rank correlation coefficient. Statistical analyses were performed in R, and significance was set at p < 0.05.

### 2.10 Data availability

Sequencing data have been deposited in public repositories (accession numbers to be provided upon publication). Proteomic datasets are included in the **Supplementary Material** or are available from the corresponding author upon reasonable request.

## 3 Results

### 3.1 Structural organization and diversification of the leishmanolysin-like multigene family

The final transcriptomic dataset consisted of ten RNA-seq libraries representing four stages of the early host–parasite interaction (0, 1, 2 and 4 h post-infection). *De novo* transcriptome assembly using Trinity generated 33,494 transcripts grouped into 26,688 putative genes, with a total assembled length of 34.1 Mb and an N50 value of 1,453 bp. Transcript lengths ranged from 190 bp to 14,183 bp. This assembly was subsequently used as the reference dataset for transcript abundance estimation and downstream analyses. The RNA-seq experiment was designed to include three biological replicates for each infection time point (0, 1, 2 and 4 h post-infection). Following library quality control, two libraries corresponding to the 4 h condition did not yield sufficient sequencing material and were excluded from subsequent analyses. Library preparation and sequencing were performed by ZF-Genomics (Leiden, The Netherlands) using the Illumina HiSeq2500 platform.

**Figure 1:**
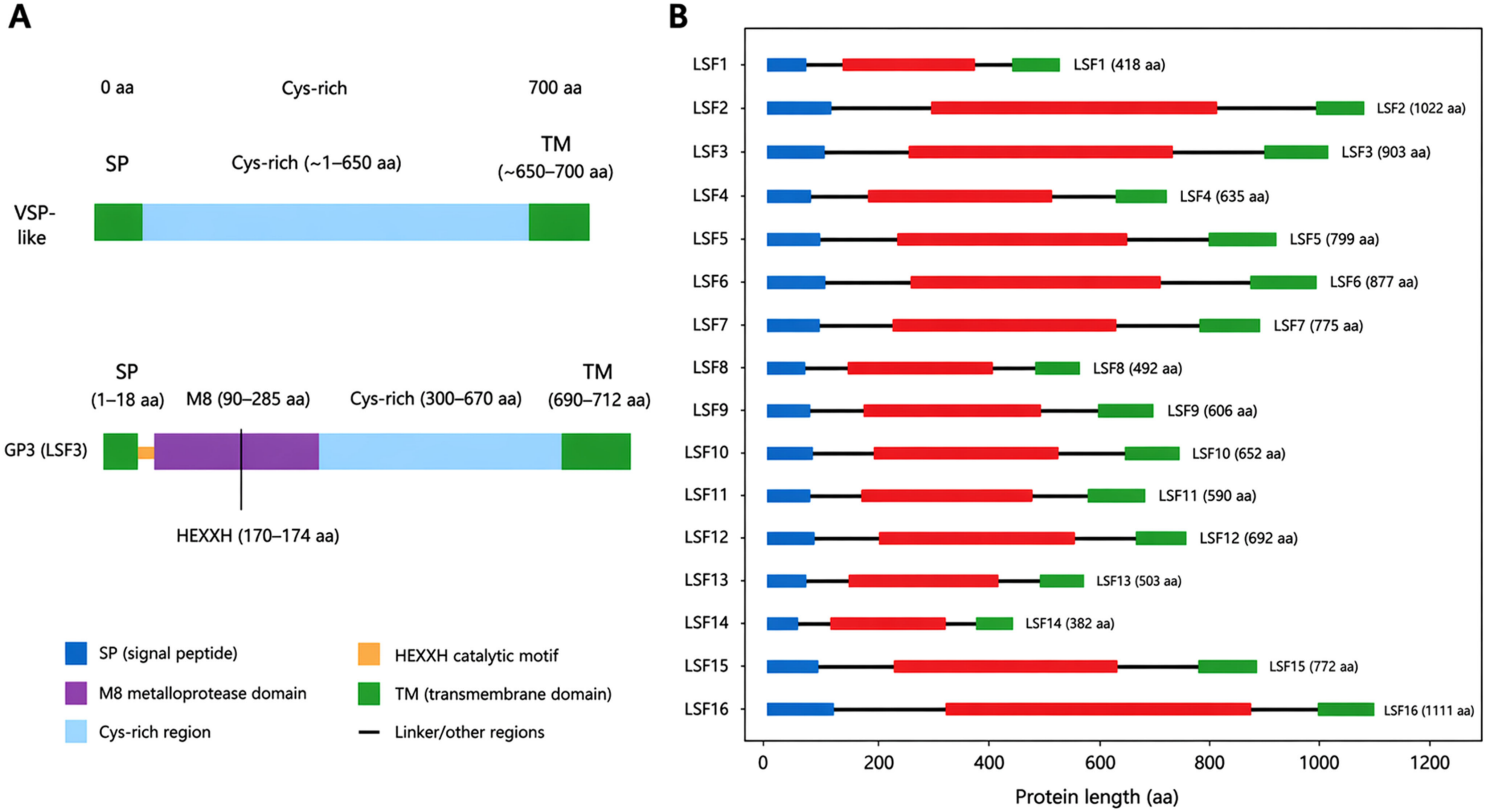
Structural organization and domain architecture of the leishmanolysin-like multigene family (LSF1–LSF16) in *Philasterides dicentrarchi*. **(A)** Schematic representation of a canonical VSP-like protein compared with a representative GP63-like (LSF) protein, highlighting key domains including the signal peptide (SP), M8 metalloprotease domain with the conserved HEXXH catalytic motif, cysteine-rich regions, and transmembrane domain (TM). **(B)** Domain organization and protein length distribution of individual LSF members (LSF1–LSF16). Blue boxes indicate signal peptides, red boxes correspond to the catalytic/metalloprotease region, and green boxes represent C-terminal transmembrane domains. Black lines indicate intervening regions, including cysteine-rich domains. Protein lengths (amino acids) are indicated for each sequence. The overall architecture reveals a conserved proteolytic core combined with variable cysteine-rich regions, supporting functional diversification within the family.

A total of 16 leishmanolysin-like proteins (LSF1–LSF16) were identified in *Philasterides dicentrarchi*, displaying conserved structural features characteristic of GP63-like metalloproteases alongside notable variability in domain architecture and protein length. All LSF proteins exhibited a common organization including an N-terminal signal peptide (SP), followed by a central M8 metalloprotease domain containing the conserved HEXXH catalytic motif, typically located within residues 90–285. Downstream of this domain, most proteins displayed extended cysteine-rich regions and a C-terminal transmembrane domain (TM), consistent with membrane anchoring and surface localization (**Figure 1A**; **Supplementary Table 1**). Despite this conserved architecture, substantial heterogeneity was observed among LSF members, with protein lengths ranging from approximately 382 to 1111 amino acids. This variability was mainly associated with differences in cysteine-rich regions and C-terminal domains (**Figure 1B**; **Supplementary Table 1**), suggesting diversification following gene duplication and potential functional specialization. A detailed representation of domain architecture and structural features across all LSF proteins is provided in **Supplementary Figure 1**. Comparative analysis with canonical GP63-like proteins confirmed conservation of key metalloprotease features alongside lineage-specific adaptations, particularly the expansion of cysteine-rich domains (**Figure 1A**).

### 3.2 Evolutionary relationships and transcriptional dynamics of the LSF gene family

**Figure 2:**
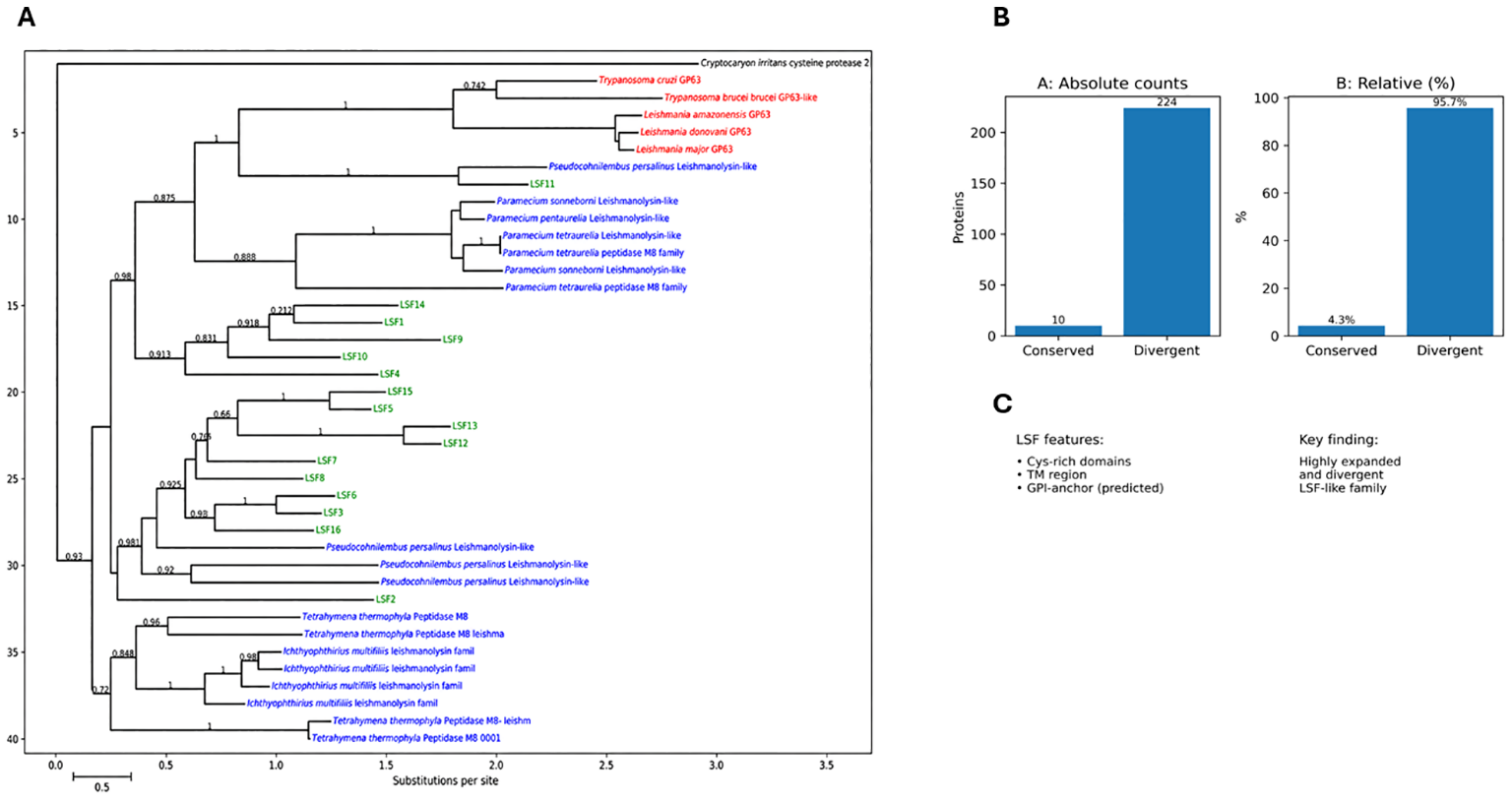
Phylogenetic diversification and structural features of leishmanolysin-like (GP63/M8) proteins in *Philasterides dicentrarchi*. **(A)** Maximum likelihood phylogenetic tree showing the relationships between leishmanolysin-like proteins identified in *Philasterides dicentrarchi* (LSF1–LSF16; highlighted in green), homologous M8 metalloproteases from representative ciliates (*Paramecium tetraurelia, Tetrahymena thermophila, Ichthyophthirius multifiliis*, and *Pseudocohnilembus persalinus*; highlighted in blue), and kinetoplastid GP63 proteins (*Leishmania* spp. and *Trypanosoma* spp.; highlighted in red). Protein sequences were aligned using MAFFT, and phylogenetic trees were reconstructed using the maximum-likelihood approach implemented in IQ-TREE, with automatic substitution-model selection performed using ModelFinder. Branch support values are indicated at nodes (SH-aLRT/ultrafast bootstrap support), and only values ≥0.70 are shown. The tree was midpoint-rooted for visualization. Branch lengths are proportional to the number of substitutions per site. A phylogenetic scale bar indicating evolutionary distance is shown in the figure. LSF proteins are distributed across multiple well-supported clades, indicating lineage-specific diversification within ciliates and clear separation from kinetoplastid GP63 proteins. **(B)** Absolute and relative distribution of conserved and divergent LSF-related sequences identified through genome-wide screening. Most sequences correspond to divergent variants, indicating extensive expansion and diversification of the LSF repertoire beyond the core conserved set. **(C)** Summary of structural features characteristic of LSF-like proteins, including cysteine-rich domains, transmembrane regions, and predicted GPI-anchor signals, highlighting their potential surface localization and functional specialization. LSF proteins are distributed across multiple well-supported clades, indicating lineage-specific diversification within ciliates and a clear evolutionary separation from kinetoplastid GP63 proteins.

**Figure 3:**
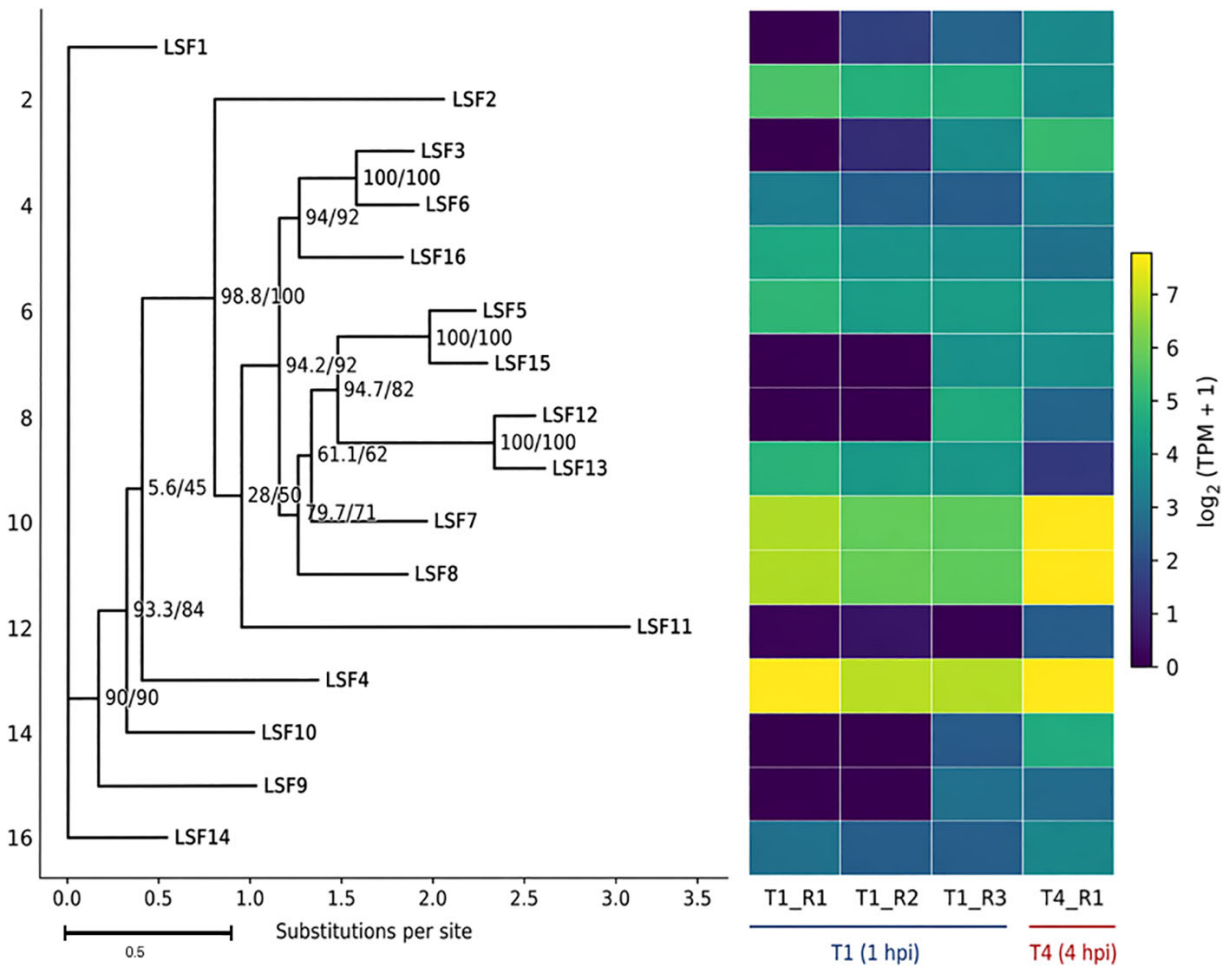
Integrated phylogenetic analysis and temporal RNA-seq expression of LSF proteins in *Philasterides dicentrarchi*. (**(A)** Maximum likelihood phylogenetic tree of the 16 leishmanolysin-like proteins (LSF1–LSF16), constructed based on amino acid sequences. Branch support values (SH-aLRT/ultrafast bootstrap) are indicated at key nodes. The scale bar represents the number of substitutions per site. **(B)** Heatmap showing representative expression profiles of LSF transcripts at 1 h and 4 h post-infection, represented as log_2_(TPM + 1). Complete TPM values for all infection stages (0, 1, 2 and 4 hpi) are provided in **Supplementary Table 2**. Columns correspond to RNA-seq samples grouped by time post-infection: T1 (1 hpi; biological replicates R1–R3) and T4 (4 hpi; biological replicate R1). Rows correspond to individual LSF genes. Color intensity reflects expression levels from low (purple/blue) to high (yellow).

To investigate the earliest transcriptional responses induced by host exposure, trophonts were recovered at 1, 2, and 4 h post-infection, representing immediate, intermediate, and early adaptive phases of parasite–host interaction. To place the LSF repertoire in an evolutionary context, phylogenetic analysis including representative GP63/M8 metalloproteases from ciliates and kinetoplastids demonstrated that the P. dicentrarchi sequences form a distinct ciliate-specific lineage clearly separated from kinetoplastid GP63 proteins (**Figure 2A**), supporting an independent evolutionary trajectory. Genome-wide screening further revealed a large repertoire of conserved and divergent LSF-related sequences (**Figure 2B**), while structural analyses identified characteristic features associated with surface localization, including cysteine-rich regions, transmembrane domains, and predicted GPI-anchor signals (**Figure 2C**). A more detailed phylogenetic reconstruction restricted to the 16 annotated LSF proteins (LSF1–LSF16) revealed diversification into several well-supported subclades (**Figure 3A**). Multiple internal nodes showed high statistical support (bootstrap/SH-aLRT values frequently >90), indicating robust evolutionary relationships within the family (**Supplementary Figure 2**). Closely related paralogous pairs such as LSF3–LSF6 and LSF5–LSF15 clustered with maximal support, suggesting relatively recent duplication events, whereas other members occupied more divergent positions within the phylogeny.

**Figure 4:**
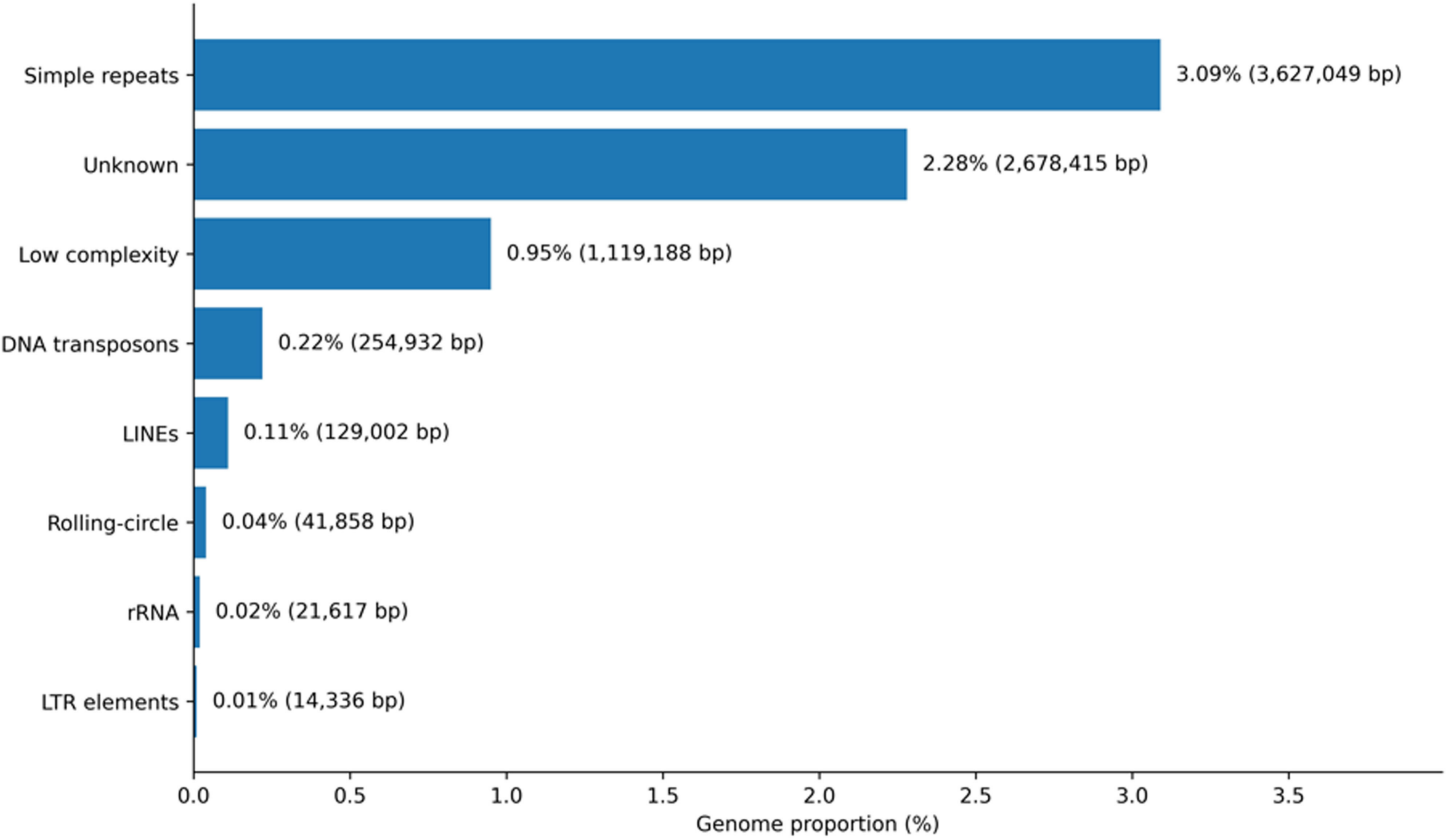
Repeatome composition of *Philasterides dicentrarchi*. Genome-wide distribution of repetitive elements identified using RepeatMasker (31). The repeat landscape is dominated by simple repeats (3.09%) and unclassified elements (2.28%), followed by low-complexity regions (0.95%), whereas canonical transposable elements are scarce. These include DNA transposons (0.22%), LINEs (0.11%), rolling-circle elements (0.04%), rRNA-derived repeats (0.02%), and LTR elements (0.01%). Values represent genome proportion and base pair content. The predominance of simple and lineage-specific repeats, together with the low abundance of transposable elements, suggests that genome plasticity in *P. dicentrarchi* is primarily driven by localized sequence features, which may contribute to the diversification of surface-associated gene families such as the leishmanolysin-like repertoire.

**Figure 5:**
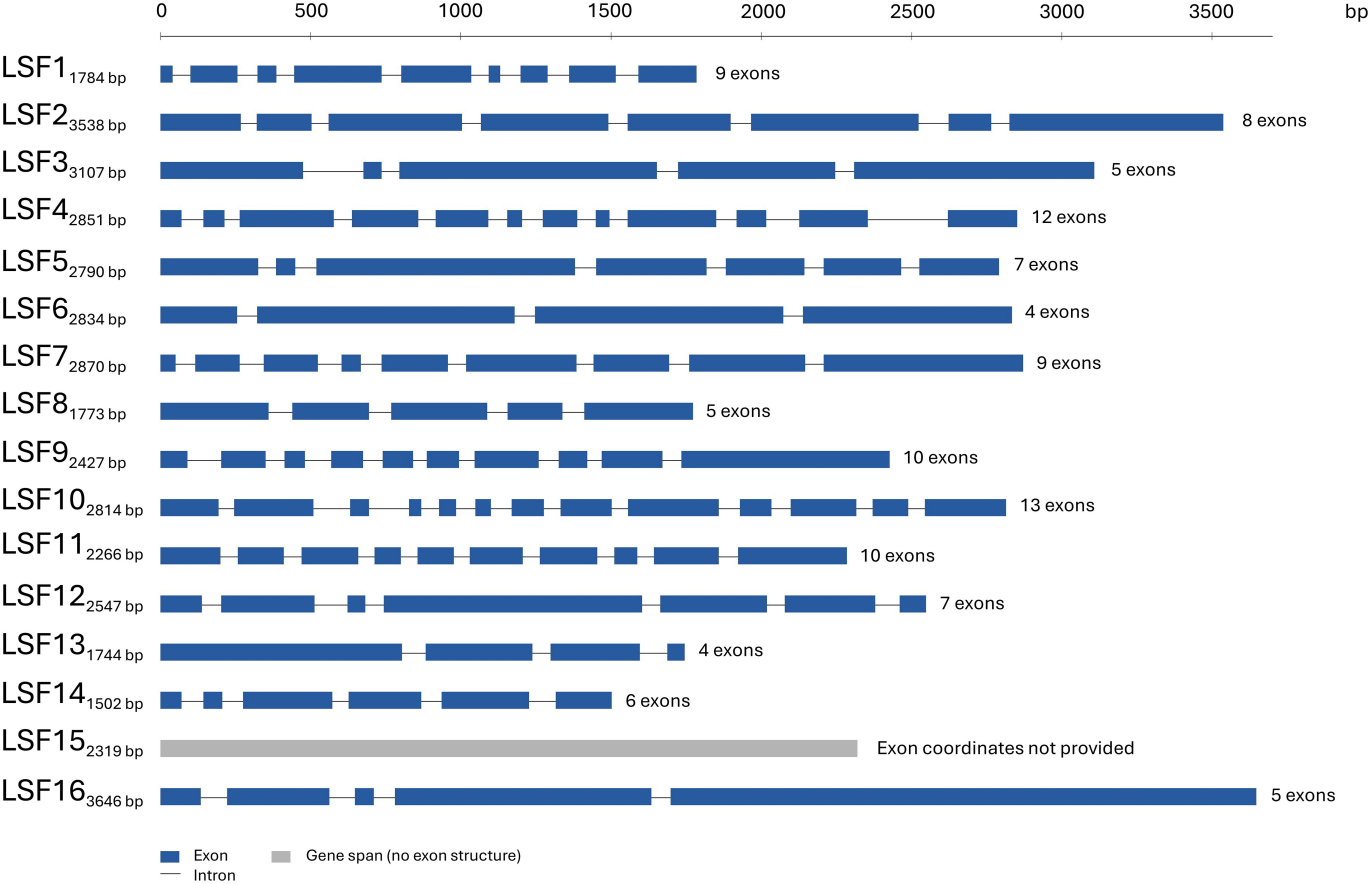
Genomic organization of leishmanolysin family genes (LSF1–LSF16) in *Philasterides dicentrarchi*. Exon–intron structures were reconstructed based on genomic mapping of coding sequences. Exons are represented as blue boxes and introns as connecting lines. Gene lengths (bp) are indicated on the left, and the total number of exons per gene is shown on the right. All structures are drawn to scale relative to genomic length. LSF genes display considerable structural variability, ranging from 4 to 13 exons, reflecting diversification within the gene family. LSF15 is represented as a continuous grey bar because exon–intron boundaries could not be resolved from the available data. The genomic span for each gene is shown proportionally along a common coordinate axis (bp).

Integration of RNA-seq data revealed marked heterogeneity in transcriptional profiles during early infection (**Figures 3B**, **4**, **5**). Most LSF genes exhibited strong upregulation at 1 h post-infection (hpi), followed by a progressive decline at later time points. Highly expressed genes included LSF7, LSF8, and LSF4, whereas other members displayed lower or more restricted expression patterns. Notably, closely related paralogs frequently exhibited distinct temporal expression profiles, whereas more distantly related members occasionally showed similar transcriptional responses. These observations indicate that phylogenetic relatedness alone does not predict expression behavior during infection and support substantial functional divergence within the LSF repertoire. No significant association was observed between phylogenetic proximity and similarity of transcriptional profiles.

### 3.3 Repeat content and genomic plasticity of the *P. dicentrarchi* genome

Genome-wide repeat annotation identified a total of 114,640 repetitive elements, representing 6.53% of the genome. The repeatome is dominated by simple repeats (3.09%) and unclassified elements (2.28%), followed by low-complexity regions (0.95%), whereas canonical transposable elements are comparatively scarce (**Figure 4**). Minor contributions from DNA transposons, LINEs, rolling-circle elements, LTR retrotransposons, and rRNA-derived repeats were also detected. This distribution indicates that the P. dicentrarchi genome exhibits a compact architecture in which localized sequence features, rather than large-scale transposon-driven expansion, represent the main source of genomic plasticity. The high abundance of simple and unclassified repeats suggests an active or historically active repeat landscape capable of promoting genomic rearrangements and duplication events. In this context, these features may contribute to the structural diversification of multigene families, including the leishmanolysin-like (LSF) repertoire.

### 3.4 Multilevel expansion, genomic organization and functional deployment of the LSF gene family

**Figure 6:**
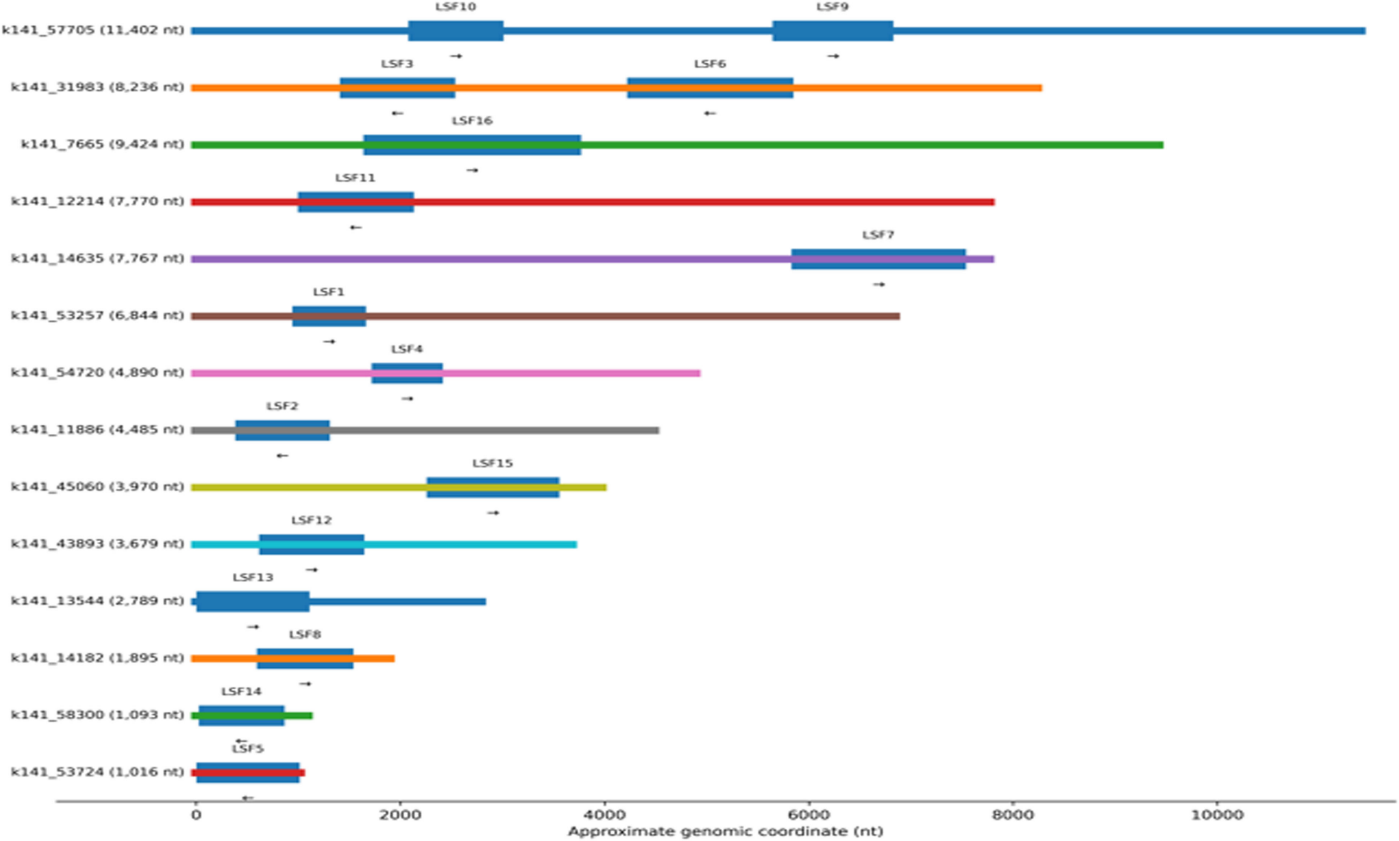
Approximate genomic distribution of leishmanolysin-like genes in *Philasterides dicentrarchi*. Approximate genomic loci of LSF1–LSF16 inferred by six-frame translated matching against the *P. dicentrarchi* genome assembly using the ciliate genetic code. Each horizontal line represents a genomic contig, and colored boxes indicate the approximate position of each mapped LSF locus. Arrow direction denotes strand orientation. Most LSF genes were distributed across different contigs, whereas two contigs (k141_31983 and k141_57705) contained two mapped loci each, suggesting local expansion or physical linkage of specific leishmanolysin-like genes. Coordinates should be interpreted as approximate loci rather than exon-resolved gene models.

To contextualize the genomic environment in which LSF genes are embedded, we first analyzed the repeat landscape of the genome (**Figure 5**). Gene structure analysis revealed substantial heterogeneity in exon–intron organization (**Figure 5**), with gene lengths ranging from ~1.5 kb to 3.6 kb and exon numbers varying between 4 and 13. Partial conservation among paralogs suggests shared ancestry, whereas variability indicates exon gain/loss events. Genomic distribution analysis showed that LSF genes are predominantly dispersed across contigs (**Figure 6**), with only limited clustering (e.g., LSF3–LSF6 and LSF9–LSF10), suggesting local duplication combined with broader genome rearrangement.

**Figure 7:**
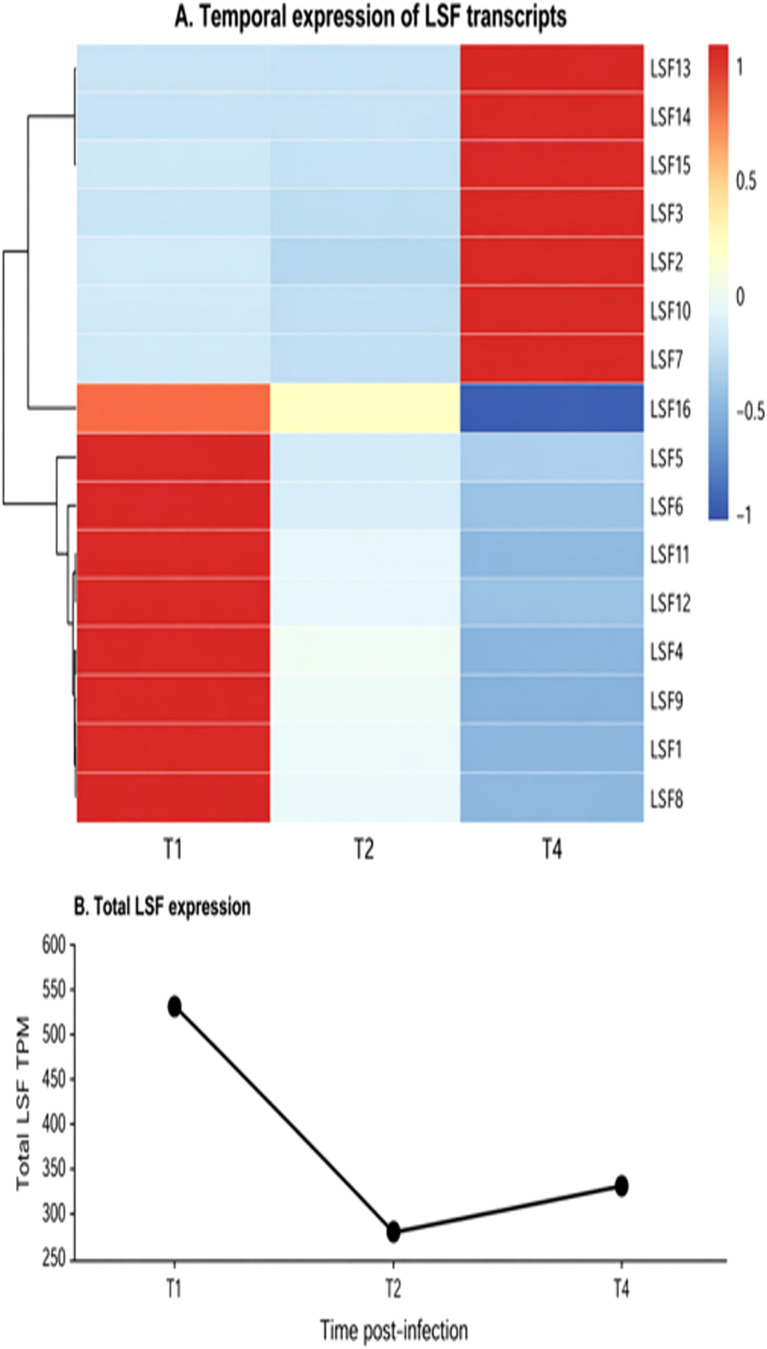
Temporal expression dynamics of leishmanolysin-like (LSF) transcripts during early infection. **(A)** Heatmap showing relative expression changes (log_2_-transformed and row-scaled TPM values) of LSF genes across infection time points (T1, T2, and T4). Genes are hierarchically clustered based on expression profiles. The color scale represents relative expression levels, from downregulation (blue) to upregulation (red). **(B)** Total LSF expression (sum of TPM values across all LSF genes) at each time point, illustrating a marked decrease at T2 followed by partial recovery at T4.

**Figure 8:**
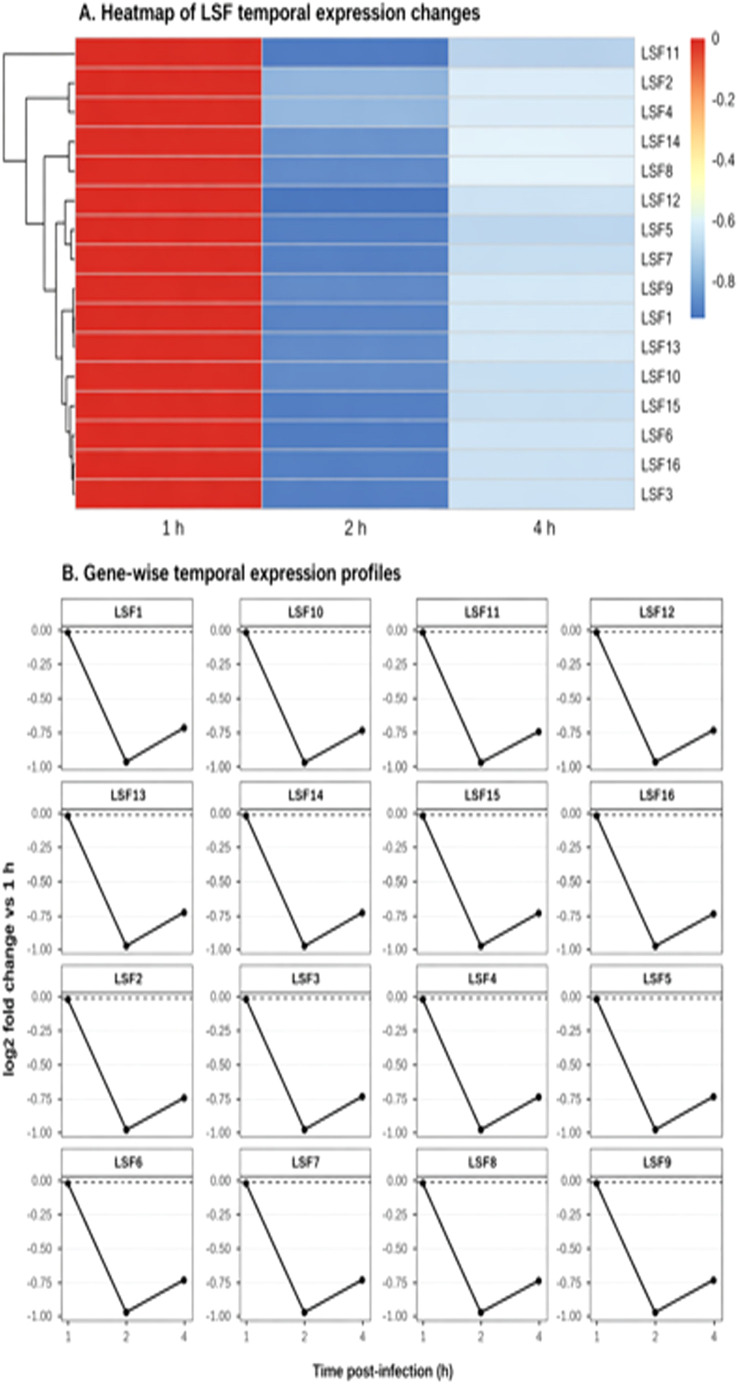
Temporal expression dynamics of leishmanolysin-like (LSF) genes during early infection. **(A)** Heatmap showing relative expression changes of LSF transcripts at 1 h, 2 h, and 4 h post-infection. Expression values were log_2_-transformed and row-scaled. Genes were hierarchically clustered according to their temporal expression profiles. The color scale indicates relative expression levels, from higher expression (red) to lower expression (blue). **(B)** Gene-wise temporal expression profiles of individual LSF genes, represented as log_2_ fold change relative to 1 h. All LSF genes exhibit a consistent pattern of downregulation at 2 h followed by partial recovery at 4 h.

RNA-seq analysis revealed marked heterogeneity in transcriptional profiles during early infection, with most LSF genes exhibiting peak expression at 1 h post-infection followed by a general decrease at later time points (**Figures 7**, **8**). The complete transcriptomic dataset, including TPM values for all biological replicates, is provided in **Supplementary Table 2**. Proteomic analysis of the surface-associated fraction (SAF) confirmed the expression of 11 out of 16 LSF proteins (**Figure 5**), with several members among the most abundant proteins detected. A significant positive correlation between transcript and protein levels (Spearman’s ρ = 0.62, p < 0.01) supports functional relevance. The complete list of identified proteins, including peptide counts, sequence coverage, and functional annotations, is provided in **Supplementary Table 3**.

### 3.5 Expansion and genomic context of the leishmanolysin-like repertoire

**Figure 9:**
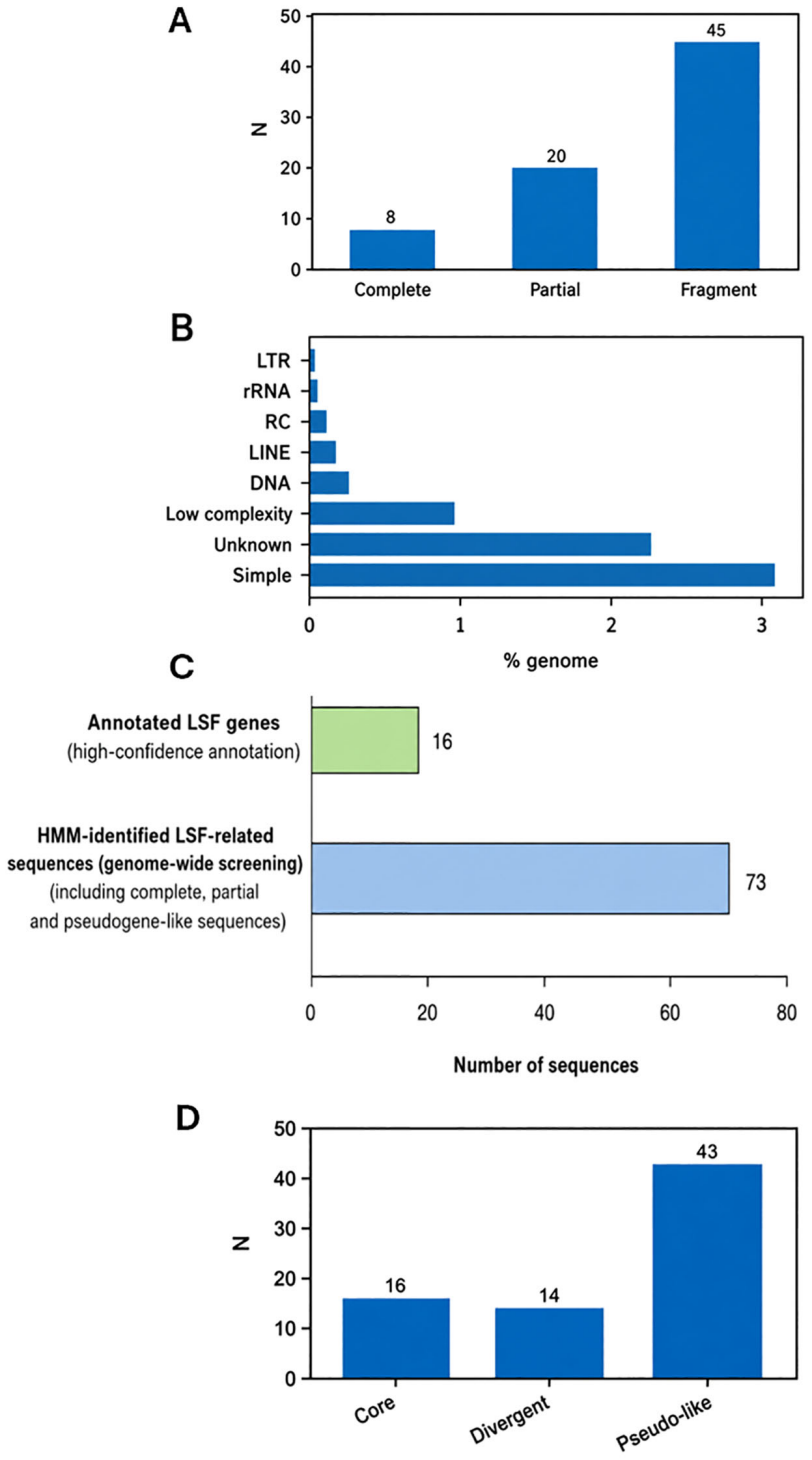
Integrated expansion and genomic context of the leishmanolysin-like (LSF) repertoire in *Philasterides dicentrarchi*. **(A)** Classification of high-confidence HMM hits (E-value < 1 × 10^-^²^0^) into complete, partial, and fragment/pseudogene-like sequences, revealing a substantial expansion of the LSF repertoire beyond the annotated set. **(B)** Genome-wide repeat composition showing the predominance of simple and unclassified repeats, consistent with a compact but structurally dynamic genome. **(C)** Comparison between annotated LSF genes (n = 16) and LSF-related sequences recovered through genome-wide HMM screening (n = 73), highlighting the extensive diversification of the LSF family beyond the repertoire identified by standard annotation pipelines. **(D)** Conceptual distribution of the LSF repertoire into core conserved genes, divergent paralogs, and pseudogene-like sequences, supporting a dynamic birth–death evolutionary model. Together, these results indicate that the LSF family in *P. dicentrarchi* is larger and more heterogeneous than previously recognized, likely shaped by duplication, divergence, and degeneration processes within a repeat-rich genomic context.

To further investigate the genomic complexity of the LSF family, we performed profile-based genome screening using a Hidden Markov Model (HMM). Classification of HMM hits based on sequence completeness revealed a heterogeneous distribution comprising complete genes, partial sequences, and fragmented or pseudogene-like elements (**Figure 9A**). The predominance of partial and fragmented sequences supports ongoing processes of duplication, divergence, and degeneration. This analysis identified a total of 73 high-confidence LSF-related sequences, substantially exceeding the 16 genes initially annotated (**Figure 9C**), indicating that standard annotation approaches underestimate the true size of the LSF repertoire.

Integration of these findings with the repeat landscape suggests that LSF expansion is closely linked to genome plasticity and may be influenced by repeat-associated recombination mechanisms (**Figure 9B**). Furthermore, classification of LSF-related sequences into core, divergent, and pseudogene-like categories revealed a predominance of non-canonical forms (**Figure 9D**), supporting a birth–death model of gene family evolution in which conserved functional genes coexist with a broader pool of evolving paralogs.

### 3.6 Proteomic validation and early functional deployment of LSF proteins

**Figure 10:**
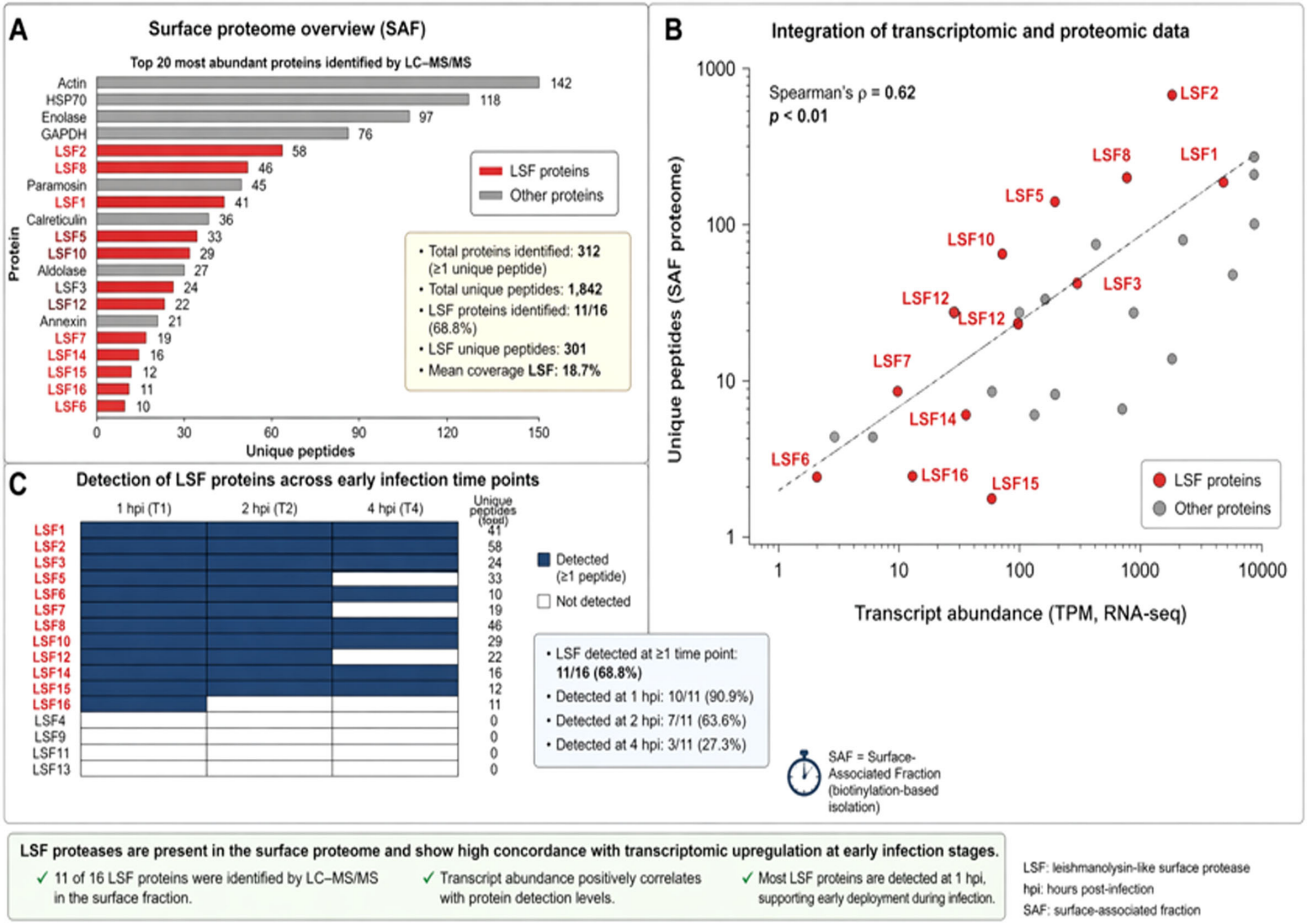
Surface proteomic identification and integration with transcriptomic data reveal the presence and early deployment of leishmanolysin-like proteases (LSFs) in *Philasterides dicentrarchi*. **(A)** Overview of the surface-associated proteome (SAF) obtained by LC–MS/MS, showing the top 20 most abundant proteins ranked by number of unique peptides. LSF proteins are highlighted in red. Summary statistics include total proteins identified (≥1 unique peptide), total unique peptides, number of LSF proteins detected, and mean coverage. **(B)** Integration of transcriptomic (RNA-seq) and proteomic data showing the relationship between transcript abundance (TPM) and protein detection (unique peptides in the SAF fraction). LSF proteins are highlighted in red, and other proteins are shown in grey. A positive correlation (Spearman’s ρ) indicates concordance between transcriptional and protein-level expression. **(C)** Detection of LSF proteins across early infection time points (1, 2, and 4 hours post-infection, hpi). Presence (≥1 unique peptide) or absence is indicated for each LSF. Most LSF proteins are detected at early stages (1hpi), with reduced detection at later time points, supporting their early deployment at the host–parasite interface. Together, these results demonstrate that LSF proteases are expressed at both transcript and protein levels, are present in the parasite surface fraction, and are predominantly deployed during early infection, consistent with a role in host–parasite immune interactions.

Proteomic analysis of the surface-associated fraction (SAF) confirmed the presence and abundance of LSF proteins at the parasite surface (**Figure 10A**; **Supplementary Table 3**). Among the top 20 most abundant proteins identified by LC–MS/MS, several LSF members (e.g., LSF2, LSF8, LSF1, and LSF5) were prominently represented. A total of 11 out of 16 LSF proteins (68.8%) were identified in the surface proteome, supporting their surface localization and functional relevance during infection. Integration of transcriptomic and proteomic datasets revealed a significant positive correlation between transcript abundance (TPM) and protein detection levels (Spearman’s ρ = 0.62, p < 0.01; **Figure 10B**; **Supplementary Table 2**), indicating that transcriptional activation is associated with protein-level expression. Temporal proteomic analysis further demonstrated that LSF proteins were predominantly identified during the earliest stages of infection, with the highest detection rate at 1 h post-infection (90.9%), followed by a decline at later time points (**Figure 10C**). This pattern is consistent with the RNA-seq data and supports rapid translation and deployment of LSF proteins during host invasion.

Together, these results demonstrate that LSF proteins are actively expressed and localized at the parasite surface during early infection, reinforcing their role in host–parasite interactions. Although LSF proteins represent only a subset of the proteins identified within the surface-associated fraction, their consistent detection across infection time points and their representation among the most abundant proteins highlight their functional importance. This enrichment, together with their coordinated transcriptional activation, supports a prominent role for LSF proteins in the early stages of host–parasite interaction.

### 3.7 Conceptual integration of surface-associated protein systems in host–parasite interaction

**Figure 11:**
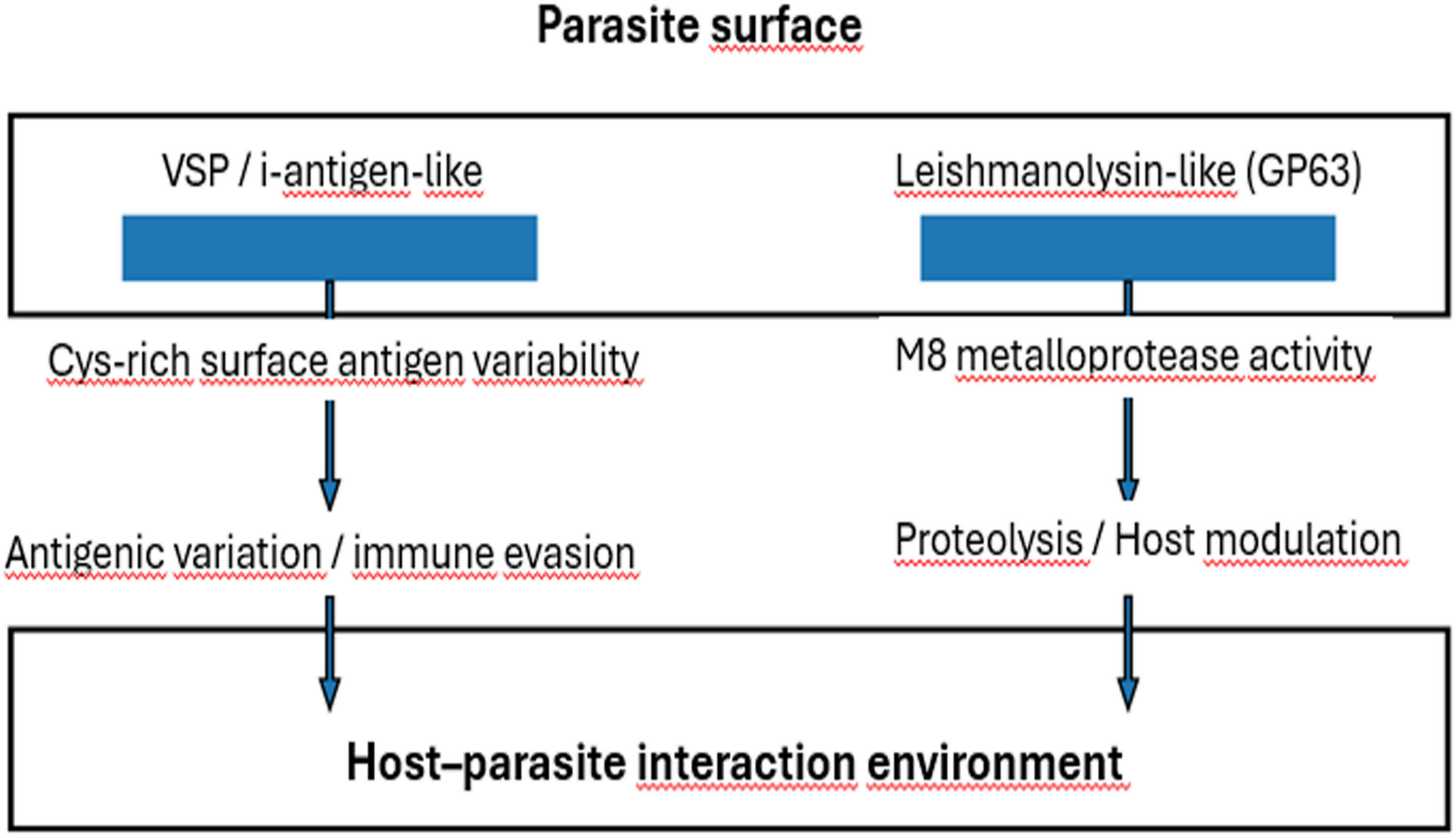
Functional model of surface-associated protein systems in *Philasterides dicentrarchi*. Schematic representation of two major classes of surface-associated proteins identified in this study. Variant surface protein (VSP/i-antigen-like) families are proposed to mediate antigenic variation through cysteine-rich domains, contributing to immune evasion. In parallel, leishmanolysin-like proteins (GP63 family) exhibit M8 metalloprotease activity, potentially involved in host protein degradation and modulation of host responses. Together, these systems define a coordinated strategy for host–parasite interaction at the parasite surface.

Based on the combined genomic, transcriptomic, and proteomic analyses, a conceptual model of surface-associated protein systems in P. dicentrarchi was established (**Figure 11**). Two major and functionally complementary classes of proteins were identified at the parasite surface: (i) VSP/i-antigen-like proteins, characterized by cysteine-rich domains and associated with antigenic variability, and (ii) LSF metalloproteases (GP63-like), associated with proteolytic activity and host modulation. VSP-like proteins likely contribute to immune evasion through continuous variation of exposed epitopes, whereas LSF proteins are predicted to mediate proteolytic modification of host components, facilitating invasion and establishment of infection. Together, these systems define a coordinated strategy at the parasite surface that integrates antigenic variation and proteolytic activity, shaping the host–parasite interaction environment.

## 4 Discussion

The present study provides a comprehensive multilevel characterization of the leishmanolysin-like (LSF) gene family in *Philasterides dicentrarchi*, integrating structural, evolutionary, genomic, transcriptomic, proteomic, and profile-based genome screening data. Collectively, these findings reveal that LSF proteins constitute a highly expanded and diversified metalloprotease repertoire that is rapidly deployed at the parasite surface during early infection, supporting a central role in host–parasite interactions.

### 4.1 Lineage-specific expansion and structural conservation

The identification of 16 LSF proteins with conserved GP63-like features, including the M8 metalloprotease domain and the HEXXH catalytic motif, indicates that these proteins retain core enzymatic functionality despite diversification. However, HMM-based genome-wide screening revealed a total of 73 high-confidence LSF-related sequences, demonstrating that the annotated gene set represents only a subset of a substantially larger repertoire (**Figure 9**). Similar expansions of metalloprotease families have been described in parasitic protozoa, where gene duplication followed by diversification enables adaptation to host environments (32). Phylogenetic analyses demonstrated that LSF proteins form a distinct ciliate-specific clade, clearly separated from kinetoplastid GP63 proteins (**Figure 2**), supporting an independent evolutionary trajectory. This observation is consistent with previous studies showing that metalloprotease families can undergo lineage-specific expansion driven by host-associated selective pressures (33, 34). The expansion of cysteine-rich domains observed in LSF proteins further suggests adaptation for extracellular stability and interaction with host molecules. Cysteine-rich surface proteins have been widely implicated in immune evasion and host interaction in protozoan parasites, including ciliates and apicomplexans (35, 36).

### 4.2 Functional divergence beyond phylogenetic relationships

A key finding of this study is the lack of strict correlation between phylogenetic clustering and transcriptional profiles. Closely related paralogs displayed divergent expression patterns, while distantly related genes exhibited similar dynamics (**Figure 3**). This decoupling suggests that regulatory evolution plays a critical role in shaping gene function. Such patterns are consistent with subfunctionalization and neofunctionalization processes following gene duplication, which have been widely reported in multigene families involved in host–pathogen interactions (37, 38). In parasitic protists, this regulatory diversification allows fine-tuned responses to environmental and host-derived signals (39). Importantly, the expanded LSF repertoire revealed by HMM analysis (**Figure 9**) suggests that this regulatory complexity likely extends beyond the annotated genes, potentially involving a broader pool of divergent paralogs and partially functional sequences.

### 4.3 Early activation and role during host invasion

The selected time points (1, 2 and 4 hpi) allowed the characterization of the immediate transcriptional activation of the LSF repertoire following host exposure and revealed that most genes reached maximal expression during the first hour of infection. RNA-seq data revealed a coordinated transcriptional program characterized by strong upregulation at 1 h post-infection, followed by a decline and partial recovery (**Figures 2**, **7**, **8**). This pattern strongly suggests that LSF proteins play a role during the earliest stages of host invasion. The concordance between transcriptional activation and proteomic detection (**Figure 10**), together with the positive correlation between mRNA abundance and protein levels, supports the functional relevance of LSF expression. Nevertheless, transcript abundance does not necessarily predict protein abundance, as post-transcriptional regulation, translational efficiency, and protein turnover may influence final protein levels. Therefore, the observed correlation should be interpreted as supportive rather than as direct evidence of quantitative coupling between transcription and translation. Early deployment of surface proteases has been described in several parasitic systems, where they contribute to tissue invasion, immune evasion, and establishment of infection (40, 41). In kinetoplastids, GP63 proteases are known to cleave host signaling molecules, complement factors, and cytoskeletal proteins, thereby modulating host immune responses (42). Although LSF proteins are evolutionarily distinct, their structural similarity suggests that they may perform analogous functions in *P. dicentrarchi*. Therefore, the observed correlation should be interpreted as supportive rather than as direct evidence of quantitative coupling between transcription and translation.

### 4.4 Genomic plasticity and diversification mechanisms

The structural organization of the LSF family provides evidence of an evolutionary history shaped by recurrent gene duplication and diversification events. The marked heterogeneity observed in exon–intron organization and genomic distribution among LSF genes suggests that both local duplication and subsequent genomic rearrangements have contributed to the expansion of this repertoire. Similar patterns have been reported for multigene families involved in host–parasite interactions in a wide range of protozoan parasites, where gene duplication generates functional redundancy while creating opportunities for adaptive diversification (33, 37). The presence of closely related paralogous genes located within the same genomic contigs is consistent with tandem or local duplication events, whereas the broader dispersal of other LSF members across the genome indicates secondary genomic reorganization. Such mechanisms have been described as major drivers of multigene family evolution in parasitic protists, including kinetoplastids and apicomplexans, where duplicated genes can subsequently acquire distinct regulatory patterns or specialized biological functions (3, 38). In the present study, the lack of a strict relationship between phylogenetic clustering and transcriptional profiles further supports the notion that regulatory divergence has accompanied structural diversification within the LSF family. Genome-wide HMM screening revealed that the annotated LSF genes represent only a fraction of a much larger repertoire composed of complete genes, divergent paralogs, and fragmented or pseudogene-like sequences. This pattern resembles the dynamic architectures described for immune-interacting multigene families in several protozoan parasites, where gene gain, diversification, and pseudogenization occur simultaneously (2, 3). Such architectures are generally interpreted as signatures of ongoing evolutionary experimentation, allowing parasites to explore novel sequence variants while maintaining a core set of functional genes.

The repeat landscape of P. dicentrarchi provides additional insight into the mechanisms underlying this diversification. In contrast to many eukaryotic genomes in which transposable elements constitute the major source of genomic expansion, the repeatome of P. dicentrarchi is dominated by simple repeats, low-complexity regions, and a substantial fraction of unclassified repetitive sequences. Similar repeat-rich environments have been associated with increased rates of recombination, local rearrangements, and gene-family diversification in several microbial eukaryotes (35, 39). Although the direct involvement of these repeats in LSF evolution remains to be experimentally demonstrated, their abundance suggests that they may provide a genomic substrate facilitating duplication and diversification processes. Collectively, the coexistence of conserved functional genes, divergent paralogs, and pseudogene-like sequences supports a birth–death model of multigene family evolution. Under this framework, repeated cycles of duplication, divergence, functional specialization, and gene loss generate a dynamic reservoir of genetic variation from which adaptive variants can emerge under changing host-associated selective pressures (33, 37). The extensive diversification observed within the LSF repertoire therefore appears consistent with a long-term evolutionary strategy that may enhance the capacity of P. dicentrarchi to modulate host interactions and adapt to fluctuating immune environments.

### 4.5 Dual strategy: antigenic variation and host modulation

The integrative model proposed in **Figure 11** suggests that P. dicentrarchi employs a dual strategy at the parasite surface, combining antigenic variation mediated by VSP/i-antigen-like proteins with active host modulation mediated by LSF proteases. Antigenic variation through cysteine-rich surface proteins is a well-established mechanism in ciliates such as Ichthyophthirius multifiliis and in other protozoan parasites, allowing evasion of host immune responses (35, 43, 44). In parallel, metalloproteases such as GP63 contribute to immune modulation by altering host signaling pathways and degrading immune effectors (42, 45). The coexistence of these two systems suggests a coordinated strategy in which structural variability limits immune recognition while proteolytic activity actively modifies the host environment. Such combined mechanisms have been described as key determinants of virulence in multiple parasitic systems (41).

### 4.6 Implications for vaccine development

The identification of LSF proteins as surface-associated, early expressed, and proteomically identified molecules highlights their potential as vaccine targets. However, the expanded repertoire revealed here suggests that targeting individual LSF members may be insufficient due to redundancy and sequence variability. In this context, conserved regions within the metalloprotease domain or structurally constrained epitopes shared among multiple LSF variants may represent more suitable targets for vaccine design. Alternatively, multivalent or epitope-based strategies could improve coverage of this diversified family. Future studies should focus on the functional characterization of LSF activity, identification of host substrates, and evaluation of immunogenicity and protective efficacy in experimental infection models.

## 5 Conclusion

In conclusion, the LSF gene family in *P. dicentrarchi* represents a highly expanded, diversified, and dynamically regulated system that integrates structural conservation with functional plasticity. The identification of a substantially larger repertoire than previously annotated, together with evidence of early activation, surface localization, and proteolytic potential, supports a central role in early host–parasite interaction and identifies LSF proteins as promising targets for future functional and immunological intervention studies. In combination with VSP-like proteins, LSF proteases define a dual adaptive strategy that likely underpins parasite virulence, immune evasion, and persistence. These findings support a model in which LSF diversification contributes to host–parasite interactions through coordinated surface deployment, immune modulation, and adaptive evolution.

## Data Availability

The RNA-seq datasets generated in this study have been deposited in the NCBI Sequence Read Archive (SRA) under BioProject accession number PRJNA1482140. The associated SRA accession numbers are currently being processed by NCBI and will be made publicly available upon completion of the processing.

## Supplementary material

Supplementary Figure 1 Domain architecture of leishmanolysin-like (LSF) proteins in *Philasterides dicentrarchi*. Schematic representation of the domain organization of the 16 LSF proteins (LSF1–LSF16). All sequences display a conserved N-terminal signal peptide (SP), followed by a central region corresponding to the M8 metalloprotease domain (GP63-like), which contains the catalytic core. Multiple internal markers indicate conserved motifs within the catalytic domain. Most LSF proteins also exhibit a C-terminal transmembrane (TM) region and a predicted glycosylphosphatidylinositol (GPI) anchor signal, consistent with membrane association and surface localization. Despite this conserved core architecture, variability in domain length and motif distribution highlights structural diversification within the family.

Supplementary Figure 2 Phylogenetic relationships within the leishmanolysin-like (LSF) gene family in *Philasterides dicentrarchi*. Phylogenetic tree showing the relationships among the 16 LSF proteins (LSF1–LSF16) inferred using a maximum likelihood approach based on aligned amino acid sequences. Branch support values (bootstrap/SH-aLRT) are indicated at the nodes. The tree reveals the presence of several well-supported subclades, highlighted in different colors, indicating diversification into distinct paralogous groups within the species. Notably, specific clusters (e.g., LSF3–LSF6–LSF16 and LSF5–LSF15) show high support values, suggesting recent duplication events, whereas more basal branches reflect deeper divergence within the family. The scale bar represents the number of substitutions per site.

Supplementary Table 1 List of genomic scaffolds identified in the *Philasterides dicentrarchi* genome assembly. Each scaffold (PZ268680–PZ268694) represents an individual assembled sequence derived from *de novo* genome reconstruction. These scaffolds were used as reference sequences for downstream analyses, including repeat annotation, gene prediction, and functional characterization of surface-associated protein families.

Supplementary Table 2 Transcriptomic profiling of leishmanolysin-like (LSF) genes during early infection in *Philasterides dicentrarchi*. **Supplementary Table 2** summarizes the transcript abundance of the 16 identified LSF genes (LSF1–LSF16) across early infection time points (1 h, 2 h, and 4 h post-infection), expressed as transcripts per million (TPM). Values are shown for each biological replicate, allowing assessment of expression consistency and variability. Overall, LSF genes exhibit a coordinated transcriptional response characterized by high expression levels at 1 h post-infection, followed by a general decrease at later time points. However, individual genes display distinct expression dynamics, reflecting functional divergence within the family. This dataset provides the quantitative basis for the temporal expression analyses presented in **Figures 4**, **5** (heatmap and temporal profiles) and supports the conclusion that LSF genes are rapidly activated during the early stages of host infection.

Supplementary Table 3 Proteomic identification of surface-associated proteins in *Philasterides dicentrarchi* and detection of leishmanolysin-like (LSF) proteins. **Supplementary Table 3** summarizes the proteins identified by LC–MS/MS in the surface-associated fraction (SAF) of *Philasterides dicentrarchi*, obtained by biotinylation-based enrichment. For each protein, the table includes the number of unique peptides, sequence coverage, and annotation based on database searches. Among the identified proteins, a subset corresponds to leishmanolysin-like (LSF) proteins, supporting their presence at the parasite surface. A total of 11 out of 16 LSF proteins were detected in the SAF, indicating a high representation of this family in the surface proteome. The quantitative proteomic data presented here provide experimental support for the transcriptomic findings and demonstrate that LSF proteins are not only transcriptionally active but also translated and exposed at the parasite surface during early infection stages.
