# Supplementary figures and images for: Evolutionary and functional dynamics of a leishmanolysin-like immune multigene family during early infection in Philasterides dicentrarchi
Source: Front Immunol. 2026 Jul 7;17:1864431. doi: 10.3389/fimmu.2026.1864431 (PMC13384842; doi:10.3389/fimmu.2026.1864431)

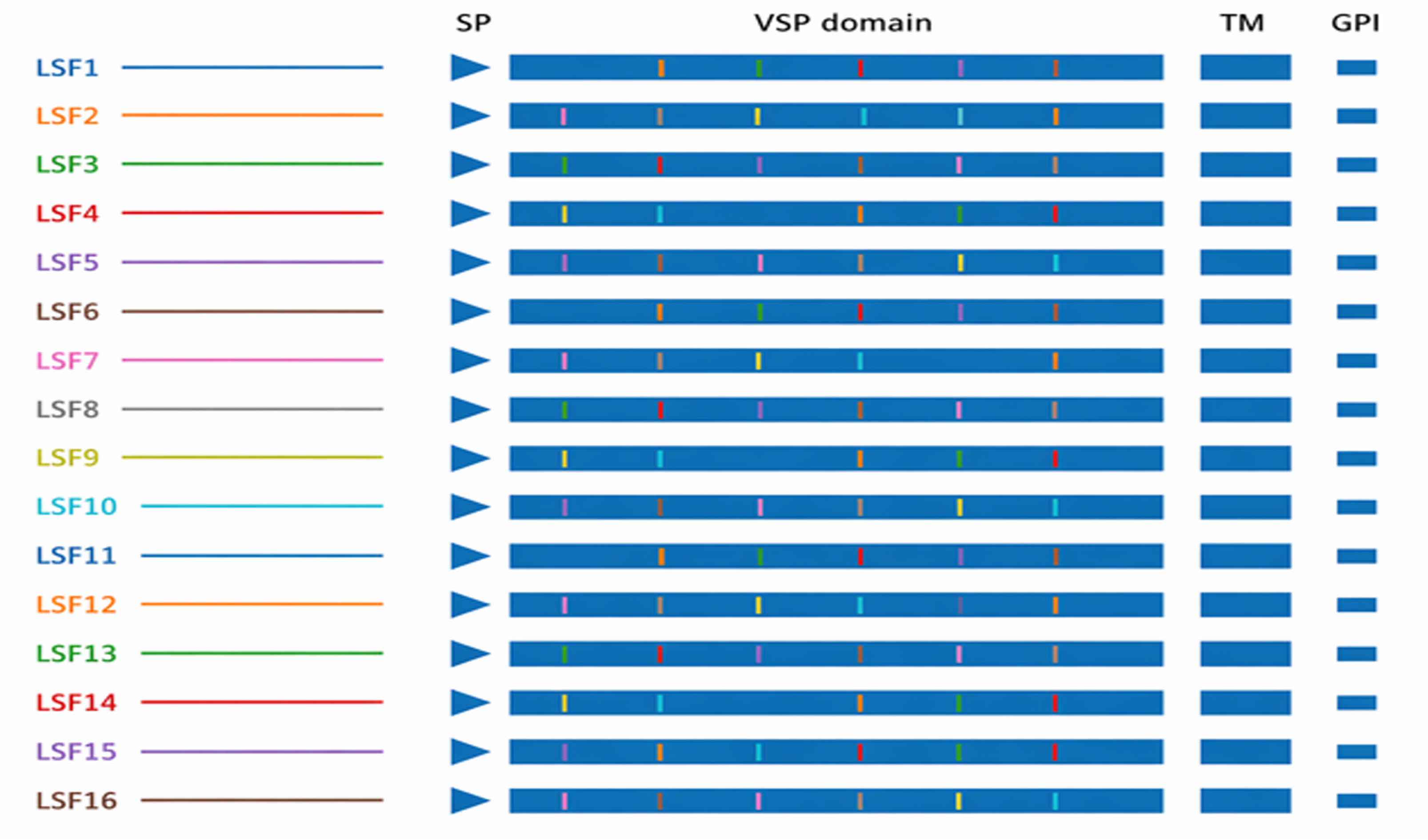

Supplement: Supplementary Figure 1 — Domain architecture of leishmanolysin-like (LSF) proteins in Philasterides dicentrarchi. Schematic representation of the domain organization of the 16 LSF proteins (LSF1–LSF16). All sequences display a conserved N-terminal signal peptide (SP), followed by a central region corresponding to the M8 metalloprotease domain (GP63-like), which contains the catalytic core. Multiple internal markers indicate conserved motifs within the catalytic domain. Most LSF proteins also exhibit a C-terminal transmembrane (TM) region and a predicted glycosylphosphatidylinositol (GPI) anchor signal, consistent with membrane association and surface localization. Despite this conserved core architecture, variability in domain length and motif distribution highlights structural diversification within the family. [file Image1.tif]

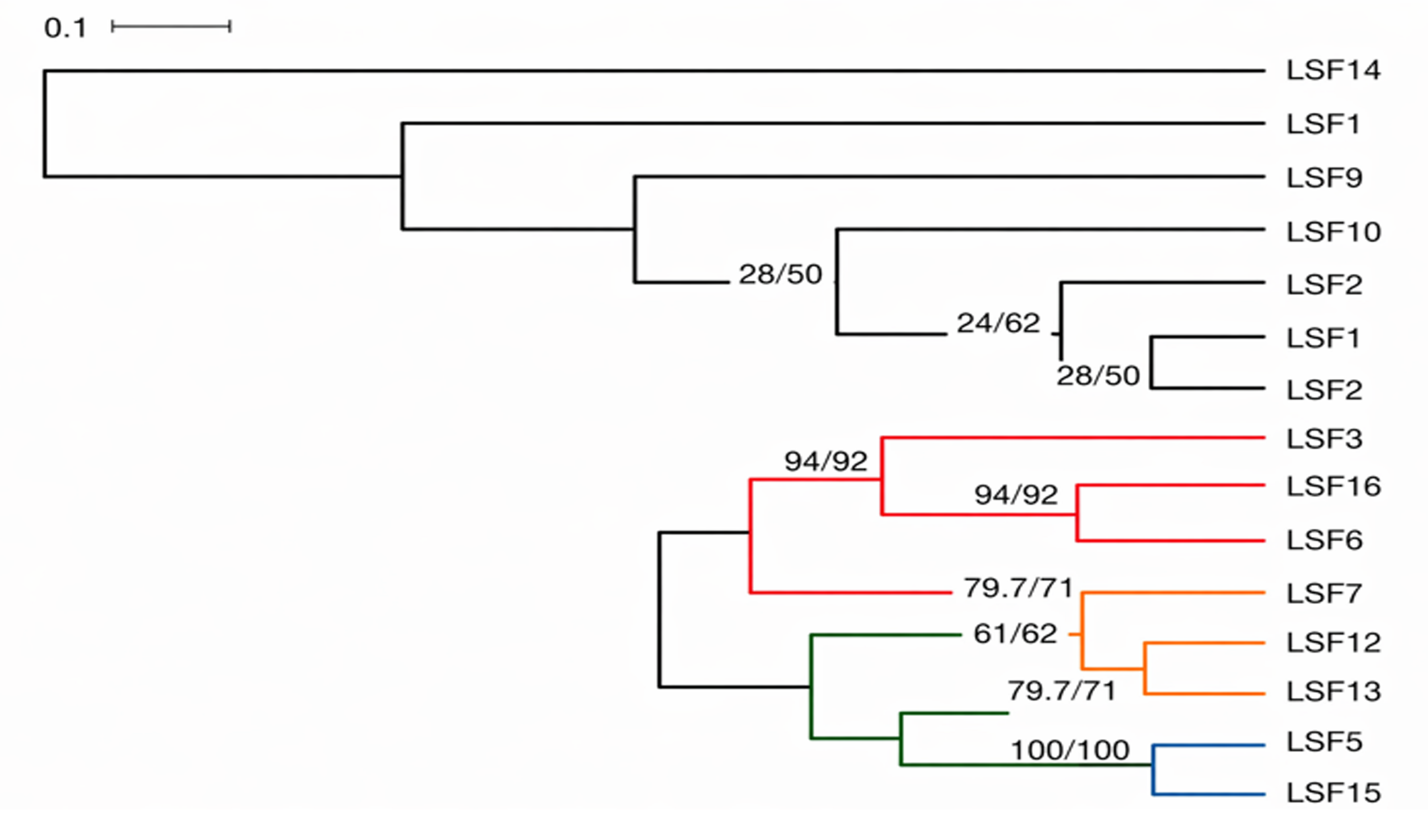

Supplement: Supplementary Figure 2 — Phylogenetic relationships within the leishmanolysin-like (LSF) gene family in Philasterides dicentrarchi. Phylogenetic tree showing the relationships among the 16 LSF proteins (LSF1–LSF16) inferred using a maximum likelihood approach based on aligned amino acid sequences. Branch support values (bootstrap/SH-aLRT) are indicated at the nodes. The tree reveals the presence of several well-supported subclades, highlighted in different colors, indicating diversification into distinct paralogous groups within the species. Notably, specific clusters (e.g., LSF3–LSF6–LSF16 and LSF5–LSF15) show high support values, suggesting recent duplication events, whereas more basal branches reflect deeper divergence within the family. The scale bar represents the number of substitutions per site. [file Image2.tif]
