# Supplementary material for: Evolutionary and functional dynamics of a leishmanolysin-like immune multigene family during early infection in Philasterides dicentrarchi
Source: Front Immunol. 2026 Jul 7;17:1864431. doi: 10.3389/fimmu.2026.1864431 (PMC13384842; doi:10.3389/fimmu.2026.1864431)
Supplement: Supplementary Table 3 — Proteomic identification of surface-associated proteins in Philasterides dicentrarchi and detection of leishmanolysin-like (LSF) proteins. Supplementary Table 3 summarizes the proteins identified by LC–MS/MS in the surface-associated fraction (SAF) of Philasterides dicentrarchi, obtained by biotinylation-based enrichment. For each protein, the table includes the number of unique peptides, sequence coverage, and annotation based on database searches. Among the identified proteins, a subset corresponds to leishmanolysin-like (LSF) proteins, supporting their presence at the parasite surface. A total of 11 out of 16 LSF proteins were detected in the SAF, indicating a high representation of this family in the surface proteome. The quantitative proteomic data presented here provide experimental support for the transcriptomic findings and demonstrate that LSF proteins are not only transcriptionally active but also translated and exposed at the parasite surface during early infection stages. [file DataSheet1.pdf]

LSF ID	Accession	Length (aa)	MW (kDa)	pI	Signal peptide	M8 domain	Cys-rich region	TM domain	GPI-anchor	Expression cluster
LSF1	PZ268680	~680	~72	5.34	Yes	Yes	Yes	Yes	No	Early
LSF2	PZ268681	~690	~73	7.92	Yes	Yes	Yes	Yes	No	Early
LSF3	PZ268682	~700	~74	5.41	Yes	Yes	Yes	Yes	No	Early
LSF4	PZ268683	~670	~71	5.46	Yes	Yes	Yes	Yes	No	Intermediate
LSF5	PZ268684	~660	~70	5.31	Yes	Yes	Yes	Yes	No	Intermediate
LSF6	QBH22559.1	~690	~73	5.42	Yes	Yes	Yes	Yes	No	Early
LSF7	PZ268685	~675	~72	5.56	Yes	Yes	Yes	Yes	No	Intermediate
LSF8	PZ268686	~685	~73	5.33	Yes	Yes	Yes	Yes	No	Late
LSF9	PZ268687	~670	~71	5.34	Yes	Yes	Yes	Yes	No	Late
LSF10	PZ268688	~660	~70	5.84	Yes	Yes	Yes	Yes	No	Late
LSF11	PZ268689	~680	~72	7.59	Yes	Yes	Yes	Yes	No	Intermediate
LSF12	PZ268690	~695	~74	5.71	Yes	Yes	Yes	Yes	No	Early
LSF13	PZ268691	~670	~71	6.03	Yes	Yes	Yes	Yes	No	Late
LSF14	PZ268692	~660	~70	5.24	Yes	Yes	Yes	Yes	No	Intermediate
LSF15	PZ268693	~685	~73	5.01	Yes	Yes	Yes	Yes	No	Early
LSF16	PZ268694	~675	~72	5.07	Yes	Yes	Yes	Yes	No	Late
